# Interplay of Institutional Infrastructure, Governance, and Cultural Values in Health System Resilience: Insights From Iran

**DOI:** 10.34172/ijhpm.9935

**Published:** 2026-05-20

**Authors:** Seyed Hasan Emami-Razavi, Sanaz Bordbar, Shiva Mafimoradi, Tahereh Rostami, Azadeh Kiumarsi

**Affiliations:** ^1^Department of General Surgery, School of Medicine, Imam Khomeini Hospital Complex, Tehran University of Medical Sciences, Tehran, Iran.; ^2^Secretariat of the Supreme Council for Health and Food Security, Ministry of Health and Medical Education, Tehran, Iran.; ^3^School of Medicine, Tehran University of Medical Sciences, Tehran, Iran.; ^4^Hematologic Malignancies Research Center, Research Institute for Oncology, Hematology and Cell Therapy, Shariati Hospital, Tehran University of Medical Sciences, Tehran, Iran.; ^5^Department of Pediatric Hematology & Oncology, School of Medicine, Children’s Medical Center Hospital, Tehran University of Medical Sciences, Tehran, Iran.

## Dear Editor,

 Over the past decade, resilience has become a central issue in global health policy, especially as health systems face pandemics, economic constraints, and complex emergencies.^1,2^ Commonly conceptualized through absorptive, adaptive, and transformative capacities, health system resilience has largely been examined through institutional and technical lenses.^2,3^ However, less attention has been paid to how resilience is formed by the interaction between governance and cultural values that influence both provider and community responses.^4^ This gap is especially relevant in contexts where social norms, belief systems, and community networks play a critical role in sustaining health service delivery under stress. In this letter, we argue that health system resilience is not only a function of institutional infrastructure, but also emerges from the dynamic interplay between institutional capacities, governance mechanisms, and cultural foundations.

 In Iran, two institutional pillars are key: a nationwide primary healthcare (PHC) network with community health workers (*behvarz*) ensuring broad population coverage and sustained access to essential care,^5^ and regionally integrated Universities of Medical Sciences that combine medical education, research, and service delivery.^6^ These structures provide stability and operational flexibility, strengthening the system’s capacity to maintain core functions under conditions of stress.

 Governance plays a central role in translating institutional capacities into effective system performance, especially under conditions of stress. In Iran, the Ministry of Health and Medical Education exercises stewardship through national policy-making, priority-setting, and resource allocation, while the Universities of Medical Sciences are responsible for implementation at provincial and local levels. This multi-level governance arrangement facilitates coordination across actors and enables context-sensitive decision-making during crises.^6,7^ By aligning resources, actors, and institutional processes, governance functions as a critical connector that converts structural capacity into real-time responsiveness and sustained service delivery.

 Cultural and social foundations represent a less visible but highly influential dimension of health system resilience takes forms. For example, during periods of stress, *behvarz* have continued home visits to monitor chronic patients despite resource shortages, motivated by a sense of moral duty. At the community level, religious gatherings were repurposed by local health authorities for health messaging, turning a potential risk into an adaptive communication channel.^8^ Community trust in *behvarz* and local health authorities may facilitate greater acceptance of service disruptions or resource constraints, such as acceptance of bed reallocation and triage decisions during surges.^3^ In addition, community-based structures, including religious and charitable organizations, can mobilize informal support, resources, and volunteers during crises, complementing formal service delivery. Additionally, neighbors have organized shared supplies, and volunteers have delivered medications to elderly households when official supply chains lagged, reflecting cultural norms of solidarity and mutual aid.

 The interaction between institutional infrastructure, governance, and cultural foundations becomes most visible during periods of crisis, where resilience is enacted in practice rather than defined in theory. For instance, during the COVID-19 pandemic, Iran’s PHC network, hospital system and governance mechanisms provided the basis for service delivery.^9^ At the same time, health workers continue service provision under pressure, alongside community cooperation and acceptance of public health measures, reflected the influence of social and cultural norms that supported system functioning.^10^

 In conclusion, the resilience of Iran’s health system emerges from the dynamics of institutional infrastructure, governance mechanisms, and culturally embedded social values. The PHC network and university-based service delivery system provide structural stability and operational flexibility, governance ensures coordination, stewardship, and adaptive decision-making, and cultural norms alongside community engagement sustain motivation, trust, and cooperation during crises.^2,3^ For policy-makers and health system leaders, Iran’s experience offers two concrete lessons. First, invest in governance mechanisms that adapt to cultural norms rather than overriding them. Second, recognize that community trust and informal solidarity are not secondary assets but core resilience capacities that can be deliberately supported, not just measured after a crisis.

## Disclosure of artificial intelligence (AI) use

 Not applicable.

## Ethical issues

 Not applicable.

## Conflicts of interest

 Authors declare that they have no conflicts of interest.
